# Homozygous inactivation of *CHEK2* is linked to a familial case of multiple primary lung cancer with accompanying cancers in other organs

**DOI:** 10.1101/mcs.a001032

**Published:** 2016-11

**Authors:** Yoji Kukita, Jiro Okami, Noriko Yoneda-Kato, Ikuko Nakamae, Takeshi Kawabata, Masahiko Higashiyama, Junya Kato, Ken Kodama, Kikuya Kato

**Affiliations:** 1 Department of Molecular and Medical Genetics, Research Institute, Osaka Medical Center for Cancer and Cardiovascular Diseases, Osaka 537-8511, Japan;; 2 Department of General Thoracic Surgery, Osaka Medical Center for Cancer and Cardiovascular Diseases, Osaka 537-8511, Japan;; 3 Department of Tumor Cell Biology, Graduate School of Biological Sciences, Nara Institute of Science and Technology, Ikoma, Nara 630-0101, Japan;; 4 Institute for Protein Research, Osaka University, Suita, Osaka 565-0871, Japan

**Keywords:** neoplasm of the lung

## Abstract

In clinical practice, there are a number of cancer patients with clear family histories, but the patients lack mutations in known familial cancer syndrome genes. Recent advances in genomic technologies have enhanced the possibility of identifying causative genes in such cases. Two siblings, an elder sister and a younger brother, were found to have multiple primary lung cancers at the age of 60. The former subsequently developed breast cancer and had a history of uterine myoma. The latter had initially developed prostate cancer at the age of 59 and had a history of colon cancer. Single-nucleotide polymorphism (SNP) genotyping revealed that ∼10% of the genomes were homozygous in both patients. Exome sequencing revealed nonsynonymous mutations in five genes in the runs of homozygosity: *CHEK2*, *FCGRT*, *INPP5J*, *MYO18B*, and *SFI1*. Evolutionary conservation of primary protein structures suggested the functional importance of the *CHEK2* mutation, p.R474C. This mutation altered the tertiary structure of CHK2 by disrupting the salt bridge between p.R474 and p.E394. No such structural changes were observed with the other mutated genes. Subsequent cell-based transfection analysis revealed that CHK2 p.R474C was unstable and scarcely activated. We concluded that the homozygous *CHEK2* variant was contributory in this case of familial cancer. Although homozygous inactivation of *CHEK2* in mice led to cancers in multiple organs, accumulation of additional human cases is needed to establish its pathogenic role in humans.

## INTRODUCTION

There are various degrees of inherited cancer susceptibility: rare high-penetrance mutations, rare disease-causing variants, and common susceptibility alleles (Fletcher and Houlston 2010). In particular, the analysis of rare high penetrance mutations has contributed to understanding the molecular mechanisms of carcinogenesis. Autosomal-dominant syndromes such as familial adenomatous polyposis and retinoblastoma have led to the discovery of tumor-suppressor genes. Hereditary cancers can be either specific to a certain organ or present in multiple organs. Examples of the former are breast cancer with *BRCA1*/*BRCA2* mutations and retinoblastoma with *RB1* mutations. An example of the latter is Li–Fraumeni syndrome caused by mutations in *TP53* (Malkin et al. 1990). Lynch syndrome is caused by defective DNA mismatch repair enzymes (Vasen et al. 1999) and usually manifests as colorectal cancer but often accompanies malignancies in other organs. However, in clinical practice, there are a number of cancer patients with clear family histories, but the patients lack mutations in known familial cancer syndrome genes. Recent advances in genomic technologies like exome and whole-genome sequencing have enhanced the possibility of identifying causative genes in such cases.

In this report, we describe the exome analysis of siblings who suffered from multiple primary lung cancer as well as cancers in other organs. The analysis revealed that homozygous inactivation of *CHEK2* was linked to the case.

## RESULTS

### Description of Patients

**Figure 1.**
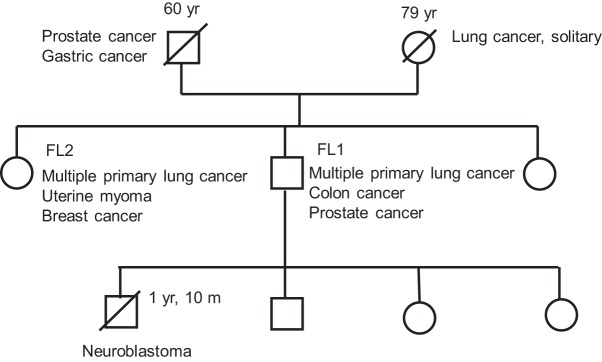
Family tree of the patients.

The family tree of the two patients is shown in Figure 1. Their parents suffered from and died of cancers in various organs. The male patient, FL1, had a history of colon cancer and developed prostate and multiple primary lung cancer with no history of smoking. At the age of 59, he was diagnosed with prostate cancer (Gleason score 4 + 3 = 7, cT2N0M0), and hormone therapy was initiated. At the age of 60, left lobectomy was performed as part of the treatment for multiple primary lung cancer. His major lesion was located at LS8, measuring 1 cm in diameter, and histological analysis revealed it to be a minimally invasive adenocarcinoma.

**Figure 2.**
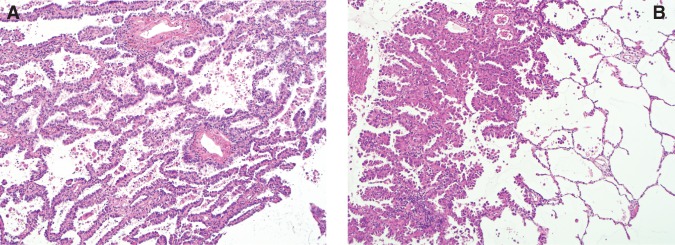
Microscopic view of multiple lung cancers. Hematoxylin and eosin staining and immunostaining with TTF1 and Nepsin A. (*A*) FL1. (*B*) FL2. Both exhibit histology of invasive adenocarcinoma.

The female patient, FL2, was 6 years older than the male patient. At the age of 38, she was diagnosed with uterine myoma and developed multiple primary lung cancer at the age of 60 with no history of smoking. Three primary tumors were located in the right lobe. The loci, diameters, and histology of these lesions were as follows: RS1, 4 cm, invasive adenocarcinoma; RS4, 1 cm, minimally invasive adenocarcinoma; and RS6, 1 cm, adenocarcinoma in situ. These lesions were surgically resected. This patient subsequently developed multiple primary lung cancer in the left lobe and breast cancer at the age of 71. The loci, diameters, and histology of the lung tumors were as follows: LS8, 2.5 cm, invasive adenocarcinoma, acinar pattern predominant; LS8, 1.9 cm, invasive adenocarcinoma, papillary pattern predominant; LS8, 2 cm, invasive adenocarcinoma, papillary pattern predominant; and LS10, 1.8 cm, invasive adenocarcinoma, papillary pattern predominant. The lung and breast tumors were surgically resected. The lung cancer *EGFR* mutation status was L858R and wild type, probably because of heterogeneity among tumor nodules. Histological and molecular analysis of the breast cancer revealed it to be an invasive ductal carcinoma with a predominant ductal component, estrogen receptor–negative, progesterone receptor–negative, and HeR2-positive. Micrographic views of both patients’ lung cancers are shown in Figure 2.

### Homozygosity Mapping

**Figure 3.**
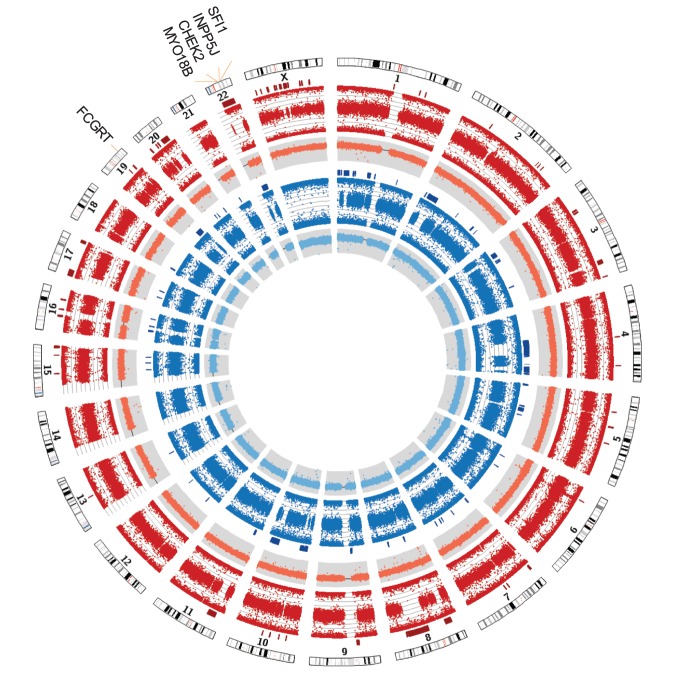
Patients’ runs of homozygosity. Patient siblings were analyzed using single-nucleotide polymorphism (SNP) arrays, and runs of homozygous genotypes were screened using PLINK. The circles, starting from outside going inward, represent the human chromosomes (Chr 1–22, X), runs of homozygosity (red bars, FL2; blue bars, FL1), B allele frequency (BAF) (red plots, FL2; blue plots, FL1), and log R ratio (LRR) (orange plots, FL2; light blue plots, FL1). The value range of the BAF plot is 0 to 1. The value range of LRR plot is −4 to 2 (“0” is marked by black lines).

We analyzed the general structures of the two patients’ genomes with single-nucleotide polymorphism (SNP) genotyping. As part of the routine analyses, we performed homozygosity mapping using the SNP-genotyping data, which involved screening for runs of homozygous genotypes in each sibling. The total lengths of homozygous segments (>1 Mb) in autosomal chromosomes were 217 and 315 Mb for FL2 and FL1, respectively (Table 1; Fig. 3). Because ∼10% of genomic regions were homozygous for both patients, we concluded that there was consanguineous marriage between their parents. Overlapping homozygous regions (63 Mb) between them were candidate regions for searching causative mutations for this family. For Chromosome X, 13 runs of homozygosity (total length 29 Mb) were detected in the female patient FL2, which were identified as possible targets. No aberrant copy-number variants were detected (Fig. 3).

**Table 1.** Runs of homozygosity

Sample	Segments	Mean (bp)	Total (bp)
FL1	65	4,850,810	315,302,625
FL2	59	4,167,608	245,888,862

Data for Chr X were not included for FL1 because he is male.

### Exome Sequencing

**Figure 4.**
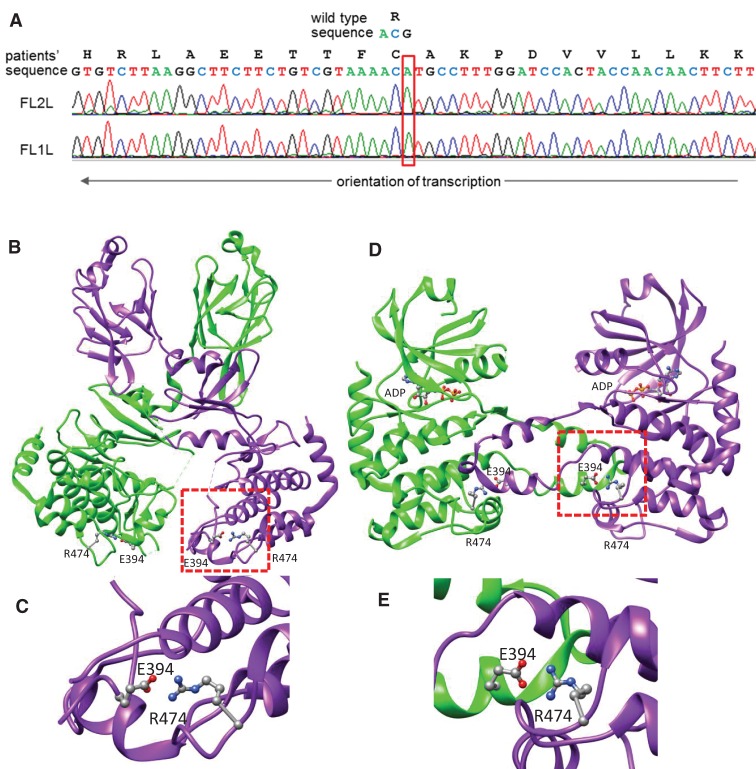
*CHEK2* mutation. (*A*) Sanger sequencing of p.R474C mutation from patient siblings. (*B*) Structure of inactive CHK2 homodimer (PDB code: 3i6w). Most residues in the activation segment are disordered and invisible. (*C*) Enlarged view of the salt bridge between p.R474 and p.E394. (*D*) Structure of active CHK2 homodimer with ADP (PDB code: 2cn5). The activation segment is visible and swapped. (*E*) Enlarged view of the interchain salt bridge between p.R474 and p.E394. The molecular graphics were generated with UCSF Chimera (Pettersen et al. 2004).

Next, to identify causative mutations in coding regions, we performed whole-exome sequencing. After we removed polymerase chain reaction (PCR)-duplicated reads from the more than 190 million reads for each sibling, we obtained sequence data on protein coding regions with a mean depth of 95× (Table 2). Approximately 10,000 variants with amino acid changes were detected in each sibling, and 80% of them were shared between the siblings (Table 3). Of the novel variants identified using public variant databases (the Database for Short Genetic Variations [dbSNP], phase 1 of the 1000 Genome Project, and the Exome Sequencing Project of the National Heart, Lung, and Blood Institute [NHLBI]), five missense variants were found in the runs of homozygosity detected above. Single missense mutations were identified in *CHEK2* (Fig. 4A), *FCGRT*, *INPP5J*, *MYO18B*, and *SFI1* (Table 4). The variants, with the exception of *MYO18B*, have been recorded in ClinVar (Landrum et al. 2016; http://www.ncbi.nlm.nih.gov/clinvar/) with low allele frequencies (< 0.03%), and their “Clinical Significance” in the database was “Uncertain significance” (*CHEK2*) or “NA” (*FCGRT*, *INPP5J*, and *SFI1*). None of them have been recorded in the Human Gene Mutation Database (HGMD) public entries (http://www.hgmd.cf.ac.uk/ac/index.php).

**Table 2.** Exome sequencing statistics

Sample	Total reads	Mapped reads	Mapping rate	On targets (bases)	Mean depth
FL1	195,020,136	191,742,948	98.3%	4,929,447,566	95.05
FL2	198,040,764	194,986,150	98.5%	4,922,943,301	94.93

**Table 3.** Variants detected by exome sequencing

Variants	FL1	FL2	Shared
Nonsynonymous SNV	9072	9212	7282
Stopgain SNV	81	80	56
Stoploss SNV	19	18	15
Splicing SNV	74	65	54
Nonframeshift insertion	82	83	61
Frameshift insertion	105	104	89
Splicing insertion	41	41	34
Nonframeshift deletion	98	96	67
Frameshift deletion	90	106	77
Splicing deletion	19	18	16
Total	9681	9823	7751

SNV, single-nucleotide variant.

**Table 4.** A list of novel homozygous variants in runs of homozygosity common to both siblings

Position^a^	Ref	Var	Allelic read depth (ref/var)		Gene symbol	RefSeq, CCDS, predicted mutation at nucleotide, and protein level^b^	SIFT^c^	PolyPhen^c^
			FL1	FL2				
Chr 19: 50027791	G	A	0/48	0/42	*FCGRT*	NM_004107.4, CCDS12770.1, c.629G>A, p.R210Q	Deleterious	Probably damaging
Chr 22: 26388338	G	T	0/96	0/67	*MYO18B*	NM_032608.5, CCDS54507.1, c.6166G>T, p.V2056L	Tolerated	Benign
Chr 22: 29090061	G	A	0/231	0/187	*CHEK2*	NM_007194.3, CCDS13843.1, c.1420C>T, p.R474C	Deleterious	Probably damaging
Chr 22: 31522910	A	G	0/26	0/21	*INPP5J*	NM_001002837.2, CCDS46687.1, c.394A>G, p.S132G	Deleterious	Benign
Chr 22: 31904305	G	A	1/60	0/73	*SFI1*	NM_001007467.2, CCDS43004.1, c.35G>A, p.S12N	Deleterious	Probably damaging

RefSeq, Reference Sequence Database; CCDS, Consensus Coding DNA Sequence; SIFT, Sorting Intolerant from Tolerant; PolyPhen, Polymorphism Phenotyping.

^a^Loci in GRCh37/hg19.

^b^A canonical transcript is indicated even if there are several alternative transcripts.

^c^The effects of variants were predicted using the Variant Effect Predictor at Ensembl (http://www.ensembl.org/info/docs/tools/vep/index.html).

### Evaluation of Candidate Genes by Primary and Tertiary Structures

First, the effects of these missense variants were predicted using the Variant Effect Predictor at Ensembl (McLaren et al. 2010), for which SIFT (Sorting Intolerant from Tolerant) (Kumar et al. 2009) and PolyPhen (Polymorphism Phenotyping) (Adzhubei et al. 2010) are used. Three variants in *CHEK2*, *FCGRT*, and *SFI1* were predicted to be “deleterious” or “probably damaging” by SIFT or PolyPhen. For these three genes, we examined whether the amino acid substitutions affected protein function based on evolutionary conservation using SIFT. *SFI1* did not have enough homologs deposited in the UniProt database and could not be subjected to the analysis. p.R474 of CHK2 (protein corresponding to *CHEK2*) was conserved in 98% of homologs. p.R218 of *FCGRT* was less conserved and appeared only in 14% of the homologs. Other amino acids such as histidine (27%) and cysteine (12%) also appeared in the homologs at this position.

Second, we examined how the amino acid substitutions affect the tertiary structure of the proteins. The tertiary structure of the inactive CHK2 homodimer (PDB code: 3i6w) is shown in Figure 4B (Cai et al. 2009). p.R474 is located away from the ATP-binding region; however, it forms a salt bridge with the well-conserved p.E394 at the end of the activation segment (Fig. 4C). This salt bridge is evolutionarily well-conserved among other kinases. However, the mutation p.R474C destroys this salt bridge and is likely to make the protein unstable (Fig. 4C). Previously, the active CHK2 homodimer structure (PDB code: 2cn5) with the swapped and ordered activation segment was reported (Fig. 4D; Oliver et al. 2006). Interestingly, the active dimer also has the salt bridge involving p.R474; however, the partner, p.E394, is provided from the other chain. This suggests that protein stability of both inactive and active states might be disturbed by p.R474C. Using prediction models, p.R474C was also predicted to be “disease causing” by MutationTaster2 (Schwarz et al. 2014) and “most likely to interfere with function” by Align GVGD (Mathe et al. 2006).

The tertiary structure of FCGRT-immunoglobulin Fc fragment complex was determined. p.R210 contacts the carboxyl terminus of the immunoglobulin Fc fragment. Although there is a salt bridge between p.R210 and the Fc fragment, the carboxyl terminus is usually not important for the overall structure of the protein. In addition, p.R210 is not well conserved, and glutamine (Q) is observed in amino acid position 210 in homologs. Thus, p.R210Q is not likely to affect the function and the structure of the protein.

The tertiary structure of SFI1 was partially determined. The amino acid substitution site is outside of the determined structure. The structure model of INPP5J was partially generated using a homologous protein (INPP5B). However, amino acid sequences of the mutated region differ. Thus, evaluating the significance of the mutations in these proteins from their tertiary structures is difficult.

### CHK2 p.R474C Protein Is Poorly Activated in the Cell upon DNA Damage

**Figure 5.**
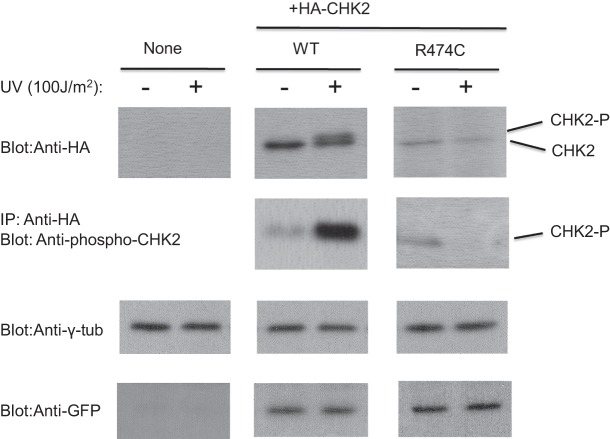
Protein expression assay of CHK2. NIH3T3 cells were mock-transfected (None) or transfected with expression vectors containing HA-tagged wild-type (WT) or mutant *CHEK2*. After treatment with or without UV radiation, cell lysates were analyzed by western blotting with an anti-HA-tag antibody (*top* panels). Alternatively, ectopic CHK2 protein was immunoprecipitated from the cell lysate with anti-HA epitope antibody and subsequently analyzed by western blotting using antiphosphorylated CHK2 antibody (second panels from *top*). CHK2-P, phosphorylated CHK2. The *bottom* panels and the second panels from the *bottom* are controls. γ-tub, γ-tubulin.

CHK2 is a cell cycle checkpoint regulator activated by DNA damage. The above analysis and the function of CHK2 suggest that *CHEK2* is a contributory gene for this familial case. We therefore examined the function of CHK2 p.R474C with a cell transfection experiment. We introduced expression vectors encoding wild-type or p.R474C *CHEK2* cDNA into NIH3T3 cells by the calcium phosphate-DNA precipitation method (Fig. 5). The expression of wild-type CHK2 protein was observed, and the protein was activated by phosphorylation after ultraviolet (UV) exposure. Unlike wild-type CHK2, p.R474C was scarcely expressed or phosphorylated regardless of UV exposure. Thus, CHK2 p.R474C was unstable and poorly activated by DNA damage in the cell.

### Variants in Other Disease-Related Genes

We surveyed variants in potential hereditary loci including those in *TP53* (causative gene for Li–Fraumeni syndrome), *BRCA2* (causative gene for hereditary breast/ovarian cancer), and mismatch repair genes (*MSH2*, *MSH6*, *PMS2*, and *EPCAM*, i.e., causative genes for Lynch syndrome), but found only common SNPs. Allele frequencies of these SNPs were 27%–100% in the Japanese population. According to ClinVar, rs1042522 (*TP53*, p.P72R) and rs169547 (*BRCA2*, p.V2466A) are “Benign” or of “Uncertain significance,” and all variants (rs1126497, rs2303424, rs1042821, rs2228006, and rs1805323) in mismatch repair genes are “Benign.” We did not detect any variants in the coding regions of lung cancer–related genes, including *YAP1* and *EGFR*. These genes are not likely affecting our patients’ phenotypes.

Among the variants detected in the runs of homozygosity common to both siblings, five were recorded as disease-associated variants in the HGMD public entries. However, they are related to diabetes or insulin secretion (rs757110 in *ABCC8*, rs5215 and rs5219 in *KCNJ11*), carotid intima media thickness (rs2468844 in *SAA2*), and oligospermia (rs11703684 in *PIWIL3*). The allele frequencies of these variants in the Japanese population are 19%–89%; therefore, they are unlikely to be involved in our patients’ phenotypes.

## DISCUSSION

CHK2 is a cell cycle checkpoint regulator activated by DNA damage. Upon DNA damage, CHK2 is phosphorylated by ataxia telangiectasia mutated (ATM), or ATM- and Rad3-related (ATR) kinases. The activated protein inhibits CDC25C phosphatase, which prevents the cell's entry into mitosis. It has also been shown to stabilize the tumor-suppressor protein p53 leading to cell cycle arrest in G_1_. In addition to this main pathway, CHK2 is involved in various other pathways inside the cell (Antoni et al. 2007; Tung and Silver 2011).

It has been debated in previous studies whether *CHEK2* was a high-penetrance cancer-causative gene. At first, *CHEK2* was claimed to be one of several causative genes for Li–Fraumeni syndrome (Bell et al. 1999). However, one of the reported *CHEK2* mutations was found to be a SNP in the duplicated region of *CHEK2* (Sodha et al. 2000), and it is now generally accepted that *CHEK2* is not a causative gene of Li–Fraumeni syndrome (Sodha et al. 2002). Mutations in *CHEK2* such as CHEK2*1100delC in the Dutch/Finnish population and p.S428F in the Ashkenazi Jew population are carried by ∼1% of people in these populations (Fletcher and Houlston 2010). In particular, CHEK2*1100delC is prevalent in the Caucasian population, and a large cohort study demonstrated that it was a rare disease-causing variant for breast cancer whose odds ratio for unselected patients was 2.7 (Weischer et al. 2008). The protein resulting from CHEK2*1100delC lacks the kinase domain, is unstable, and is associated with complete loss of expression of the protein (Bahassi el et al. 2007). However, it is difficult to demonstrate that *CHEK2* acts as a tumor-suppressor gene (i.e., functioning through somatic loss or inactivation of the wild-type allele) because of its low risk ratio and low allele frequency. It should be noted that somatic mutations in *CHEK2* are infrequent. For example, in lung cancer, the incidence of *CHEK2* mutations and copy-number variants is 0.99% (22/2219) and 0.54% (6/1112), respectively (Catalogue of Somatic Mutations in Cancer; http://cancer.sanger.ac.uk/cosmic).

Two recent studies reported on the homozygous mutation of CHEK2*1100delC (Adank et al. 2011; Huijts et al. 2014). Both studies indicated that homozygotes had increased breast cancer risk compared with the heterozygotes of CHEK2*1100delC. It is important to note that seven of 10 homozygous breast cancer patients developed multiple primary tumors (Adank et al. 2011), and three of five developed contralateral breast cancer (Huijts et al. 2014). van Puijenbroek et al. reported a sporadic case of colorectal cancer with homozygous CHEK2*1100delC deletion (van Puijenbroek et al. 2005). This patient manifested no significant clinical phenotypes, but died at the relatively early age of 52.

Unlike the Caucasian population, there are no *CHEK2* variants comparable with CHEK2*1100delC in the Asian population (Chen et al. 2008; Choi et al. 2008). The incidence of inactivating mutations is less in the Asian population. The case described here is a very rare case of homozygous inactivating mutations in *CHEK2* in the Asian population.

In the NHLBI Exome Sequencing Project (http://evs.gs.washington.edu/EVS/), heterozygous p.R474H variants (by a base change in neighboring nucleotide position of the p.R474C variant) had been detected in a European– and African–American population. Allele frequency in this database (3733 individuals) is 0.03%. During preparation of this manuscript, a British individual with a heterozygous p.R474C variant was recorded in the 1000 Genomes Project phase 3 (http://www.1000genomes.org/). Allele frequency in the 1000 Genomes Project (2503 individuals including 504 East Asian) is 0.02%. So far, no homozygous variant of p.R474 has been detected in healthy individuals.

The main question is whether the homozygous inactivation of the *CHEK2* gene constitutes a new disease entity. Although other reported cases did not manifest such strong clinical phenotypes as our case study, there was high incidence of multiple primary tumors. With regard to the sporadic case, if a suitable treatment was performed, the patient might have developed cancers in other organs (van Puijenbroek et al. 2005). Although heterozygous *CHEK2* mutations were previously denied as the cause of multiple familial cancers, these data suggest the possibility that homozygous inactivation of this protein may lead to multi-organ cancer. A mouse model in which the wild-type *Chek2* has been replaced by a CHEK2*1100delC allele exhibited a similar phenotype: mice homozygous for CHEK2*1100delC produced significantly more tumors than wild-type mice, whereas heterozygous mice were not statistically different from wild type (Bahassi el et al. 2009). The severity of the symptoms is likely to be variable for *CHEK2* mutations, and accumulation of more cases will clarify *CHEK2*’s role in cancer development.

It should be noted that the siblings’ mother had lung cancer and their father had prostate and gastric cancer and FL1's son had neuroblastoma, subsequently leading to their death. Given this family history, it is possible that there are additional contributory genes and an autosomal-dominant syndrome, which is the more common mode of inheritance in hereditary cancer syndromes. Such genes might be missed in the variant analysis because of no reliable Asian controls or small exon level copy-number variants that are not detectable by exome sequencing and SNP arrays.

## METHODS

### DNA Extraction

Genomic DNA from peripheral blood monocytes was extracted with a QIAamp DNA Mini Kit (QIAGEN). DNA concentration was determined with the use of a Qubit dsDNA HS Assay Kit (Life Technologies). DNA samples were examined by electrophoresis on 1% agarose gels to confirm a lack of significant degradation.

### Tissue Preparation

Formalin-fixed, paraffin-embedded tissue sections were prepared as part of routine medical practices. In addition to hematoxylin/eosin, the sections were stained with anti-TTF1 and anti-Napsin A.

### SNP Array Experiment

SNP array experiments were performed with an Illumina Omni1-Quad chip (which interrogates more than one million loci). Base-calling and calculation of BAF and LRR (or log_2_(*R*_observed_/*R*_expected_), where *R* is probe intensity) were done by GenomeStudio (Illumina). Because BAF is one allele frequency of two alleles, its value is around 0 or 1 for homozygotes and around 0.5 for heterozygotes. *R*_expected_ is interpolated from the observed allelic ratio with respect to the canonical genotype clusters (Peiffer et al. 2006), and LRR represents relative copy-number status of the position. For normal-copy-number regions, the value is around 0. Positive and negative values are gain and loss changes, respectively. The mean SNP call rate was >99.6%. Runs of homozygous genotypes within individuals were screened using PLINK (Purcell et al. 2007) with homozygous segment criteria: 1000 kb length, 100 SNPs, 50 kb/SNP density, and 1000 kb largest gap. The circle plot diagram shown in Figure 3 was drawn using Circos (Krzywinski et al. 2009).

### Exome Sequencing and Data Analysis

Patients’ DNA fragments of exonic regions were enriched with SureSelect Human All Exon 50 M kit (Agilent). Recovered DNA fragments were sequenced as 90-bp paired-end reads on an Illumina HiSeq 2000. Total reads obtained were 195,020,136 and 198,040,764 for patient FL1 and FL2, respectively. These exome sequencing procedures were done by BGI exome service. We aligned paired-end reads to the human reference genome (hg19) with Burrows–Wheeler alignment (BWA) (Li and Durbin 2009) and created .bam files using SAMtools (Li et al. 2009). Base call quality recalibration and local realignment were also performed using the Genome Analysis Toolkit (GATK) (DePristo et al. 2011). Sequence variants were detected by UnifiedGenotyper in GATK (DePristo et al. 2011). Variants in positions with low coverage (less than eight reads) were discarded. Annotation for detected variants was performed using ANNOVAR (Wang et al. 2010). Novel and known variants were discriminated using variant data: dbSNP build 135 (http://www.ncbi.nlm.nih.gov/SNP/), phase 1 data of 1000 Genomes Project (http://www.1000genomes.org/), and 5400 exome data of Exome Sequencing Project (https://esp.gs.washington.edu/drupal/).

### Construction of Expression Vectors and Protein Expression Analysis

We amplified *CHEK2* coding sequences from cDNAs of both a patient with the mutant gene (p.R474C) and a person with wild-type *CHEK2*, using a 5′-side primer with HA-tag, 5′-AGA TCT CTC GAG ACC ATG TAC CCA TAC GAT GTT CCA GAT TAC GCT TCT CGG GAG TCG GAT GTT GAG G-3′, and a 3′-side primer, 5′-GTT AAC GAA TTC CGG AGT TCA CAA CAC AGC AGC A-3′. *CHEK2* fragments were inserted into the XhoI–EcoRI site of pMSCVpuro (Clontech). *CHEK2* coding regions in plasmid constructs were confirmed to be same to RefSeq NM_007194.3 except p.R474C using Sanger sequencing. The plasmid constructs were transfected into NIH3T3 cells, and protein expression was analyzed as described in our previous work (Yoshida et al. 2013).

## ADDITIONAL INFORMATION

### Data Deposition and Access

The SNP-genotyping and exome sequencing data were deposited in the Japanese Genotype-phenotype Archive (JGA) under accession number JGAS00000000057 (https://ddbj.nig.ac.jp/jga/viewer/view/studies) under Type I security. The *CHEK2* variant p.R474C has been deposited in the ClinVar (http://www.ncbi.nlm.nih.gov/clinvar/) database under accession number SCV000282240.

### Ethics Statement

Written informed consent for research and data sharing was obtained from both patients. This study was approved by the ethics committee of the Osaka Medical Center for Cancer and Cardiovascular Diseases (Approval No. 1603315234).

### Acknowledgments

This work was partly supported by the Ministry of Education, Culture, Sports, Science & Technology in Japan (JSPS KAKENHI) under Grant Number 25430180, the Osaka Community Foundation, the Charitable Trust Osaka Cancer Research Foundation, and the Osaka Medical Research Foundation for Intractable Diseases (Y.K.). This work was also partly supported by the Platform for Drug Discovery, Informatics, and Structural Life Science from the Japan Agency for Medical Research and Development (T.K.).

### Author Contributions

K.Ka. and Y.K. conceived and designed the experiments. Y.K., N.Y.K., I.N., and J.K. performed the experiments. Y.K., T.K., J.K., and K.Ka. analyzed the data. J.O., M.H., and K.Ko. contributed materials/analysis tools. K.Ka. and Y.K. wrote the manuscript.

## Referees

Raymond D. Kim

Anonymous
